# A case of localized tracheobronchial relapsing polychondritis with positive matrilin-1 staining

**DOI:** 10.1186/s41927-019-0103-6

**Published:** 2020-01-29

**Authors:** Tomonori Makiguchi, Akira Koarai, Chihiro Inoue, Yayoi Aoyama, Taizo Hirano, Takashi Ohe, Tomohiro Ichikawa, Yutaka Shishikura, Hanae Komuro, Yoko Tsukita, Naoki Tode, Tadahisa Numakura, Tsutomu Saito, Teruyuki Sato, Yoshiya Mitsuhashi, Tsutomu Tamada, Hisatoshi Sugiura, Masakazu Ichinose

**Affiliations:** 1 Department of Respiratory Medicine, Tohoku University Graduate School of Medicine, 1-1 Seiryo-machi, Aoba-ku, Sendai, 980-8574 Japan; 2 Department of Anatomic Pathology, Tohoku University Graduate School of Medicine, 1-1 Seiryo-machi, Aoba-ku, Sendai, 980-8574 Japan

**Keywords:** Relapsing polychondritis, Tracheobronchial, Matrilin-1

## Abstract

**Background:**

Relapsing polychondritis (RPC) is a rare progressive autoimmune disease characterized by inflammation in the cartilage of multiple organs. Tracheobronchial involvement appears in nearly half of RPC patients during the course of their disease and represents the main cause of death. Localized tracheobronchial RPC is much rarer, and the pathogenesis remains unclear. Matrilin-1 is a non-collagenous cartilage matrix protein and has been suggested to be a potent autoantigen that induces the airway disease of RPC in animal models. However, the expression of matrilin-1 in tracheobronchial tissue in human remains unclear. Therefore, we examined the expression of matrilin-1 in the tracheal and auricular tissues in a localized tracheobronchial RPC patient.

**Case presentation:**

A 62-year-old man with systemic sclerosis presented with cough and dyspnea on exertion. The lung function test showed an expiratory flow limitation and chest computed tomography showed diffuse thickness from the trachea to the bronchiole. No other tests showed abnormal findings. To evaluate further, bronchoscopy was performed and endobronchial ultrasonography showed thickness in the fourth-marginal echo layer suggesting inflammation of the cartilage. However, the tracheal biopsy showed no specific findings. The subsequent surgical tracheal biopsies showed inflammatory cell infiltration with destruction of the cartilage. Neither auricular nor nasal deformity, except for a tracheobronchial lesion, was detected. Biopsy from the left auricular cartilage also did not show any inflammatory changes. Finally, we diagnosed the patient with localized tracheobronchial RPC. To address the hypothesis that autoimmunity against matrilin-1 is involved in the pathogenesis of localized tracheobronchial RPC, we evaluated the expression level of matrilin-1 in a tracheal and auricular specimen from this patient. Immunohistochemical staining with anti-matrilin-1 antibody showed matrilin-1 in the tracheal but not in the auricular cartilage.

**Conclusions:**

We first demonstrated the expression of matrilin-1 in tracheal but not in auricular cartilage in a localized tracheobronchial RPC patient. This result supports the possibility that matrilin-1 is involved in the pathogenesis of localized tracheobronchial RPC. However, this is only one case report and further observations will be needed to confirm this result.

## Background

Relapsing polychondritis (RPC) is a rare progressive autoimmune disease affecting cartilaginous structures including ear, eye, nose, larynx, trachea, bronchus, joints and heart valves [1, 2]. Tracheobronchial involvement appears in nearly half of RPC patients during the course of their disease and represents the main cause of death [2]. However, tracheobronchial involvement is uncommon at the onset and the diagnosis of localized tracheobronchial RPC is difficult because of the absence of typical auricular or nasal symptoms [3]. In addition, the pathogenesis of localized tracheobronchial RPC remains unclear.

Matrilin-1, one of a four-member family of oligomeric extracellular adaptor proteins [4], has been shown to be a potent autoantigen that induces the airway disease of RPC in animal models [5, 6]. In addition, increased serum levels of anti-matrilin-1 antibody and matrilin-1 from RPC patients have been reported to be correlated with the severity of respiratory symptoms [7, 8]. Until now, autoimmunity against matrilin-1 has been suggested to be involved in the pathogenesis of localized tracheobronchial RPC, but the expression of matrilin-1 in tracheobronchial tissue in human remains unclear. Therefore, we examined the expression of matrilin-1 in the tracheal and auricular tissues in a localized tracheobronchial RPC patient.

## Case presentation

**Fig. 1:**
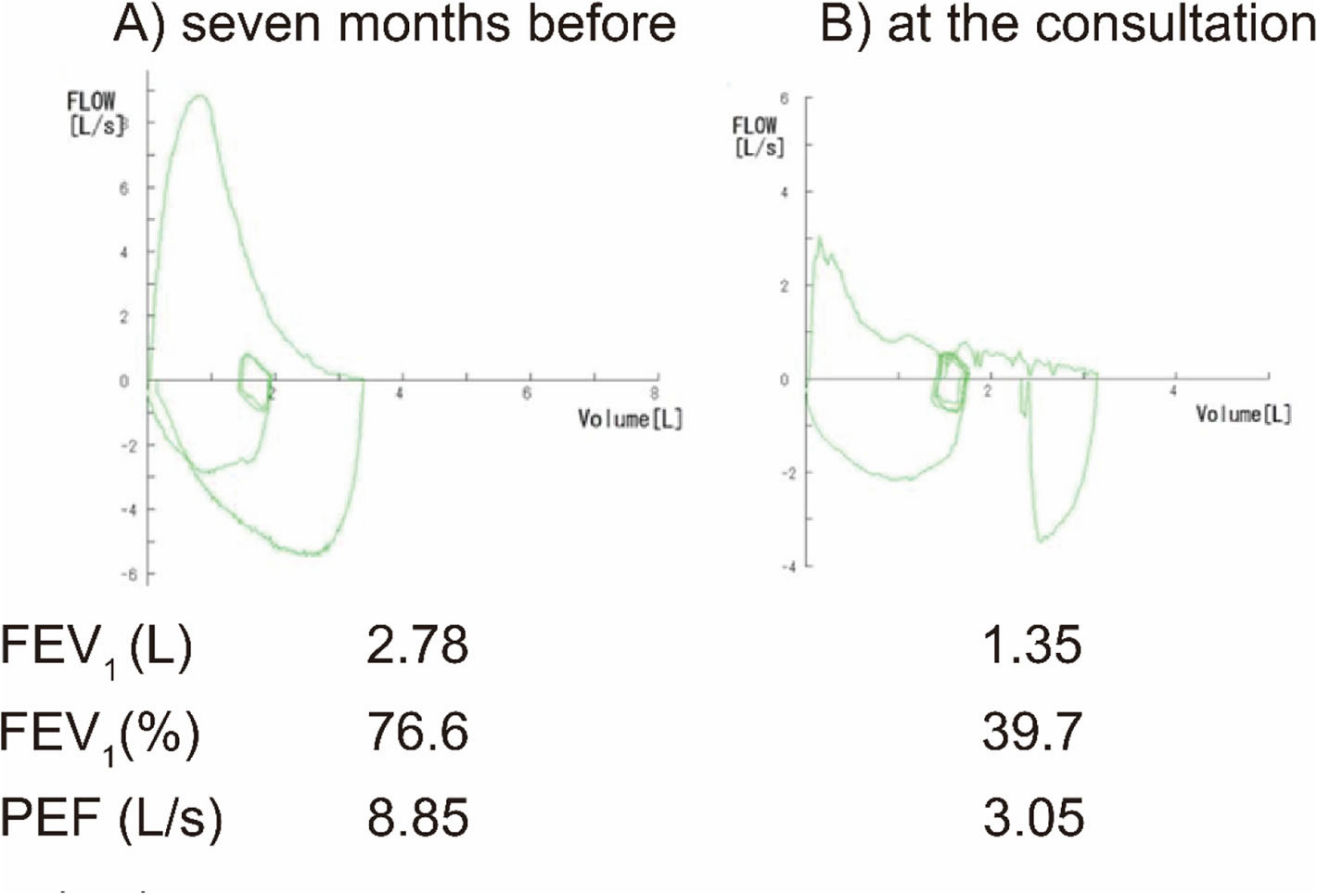
Lung function test during the clinical course. Lung function test 7 months before (**a**) and at the time of consultation (**b**). Seven months before the consultation, the flow volume curve was almost normal except for a slight downward convex in the latter half of the expiratory phase (**a**). At the time of the consultation, both peak expiratory flow (PEF) and forced expiratory flow in 1 s (FEV_1_) were markedly decreased (**b**)

**Fig. 2:**
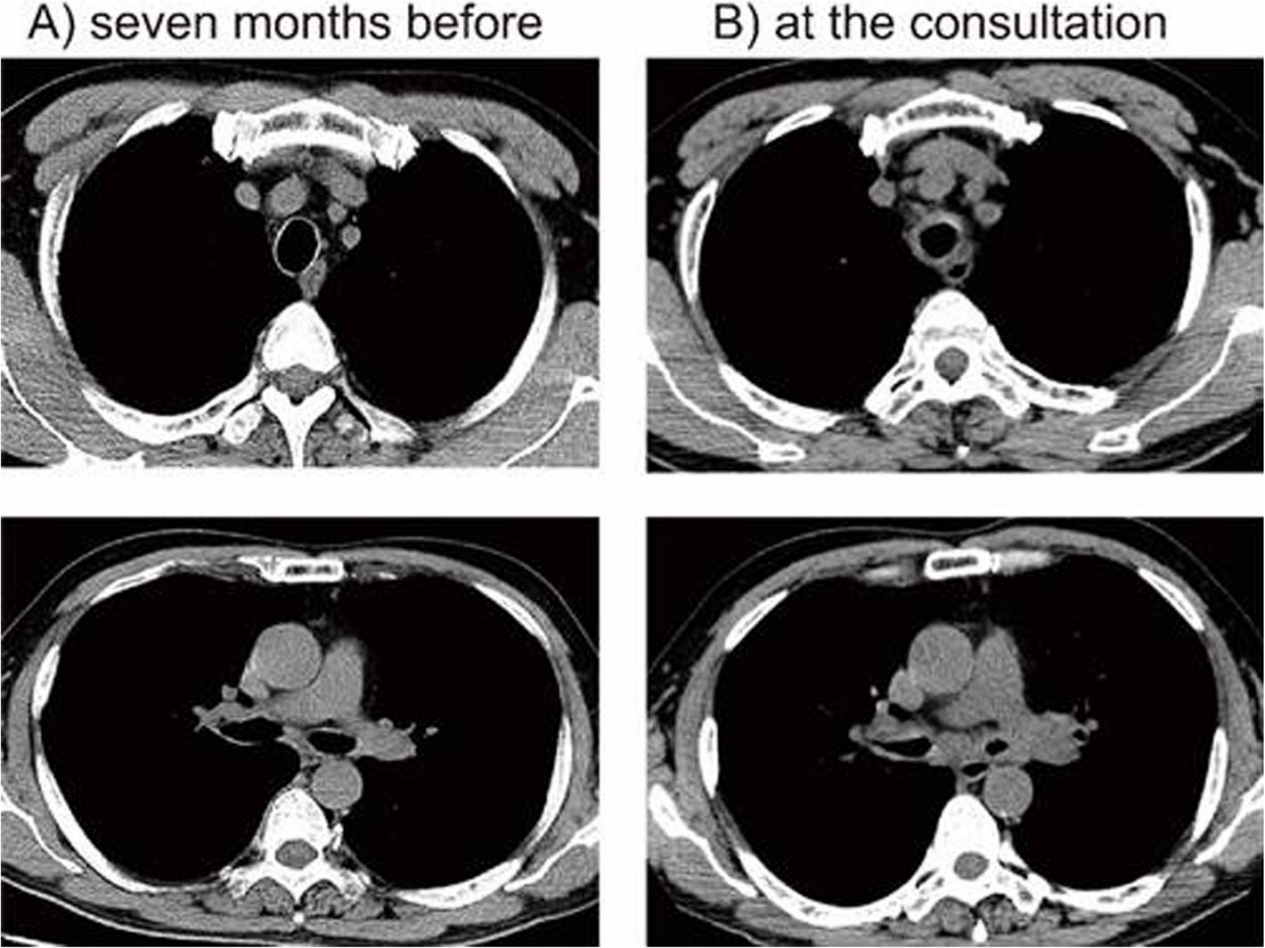
Computer tomography (CT) during the clinical course. Thoracic CT 7 months before (**a**) and at the time of consultation (**b**). Compared to before the onset, the wall of the trachea to the bronchiole had thickened diffusely

**Fig. 3:**
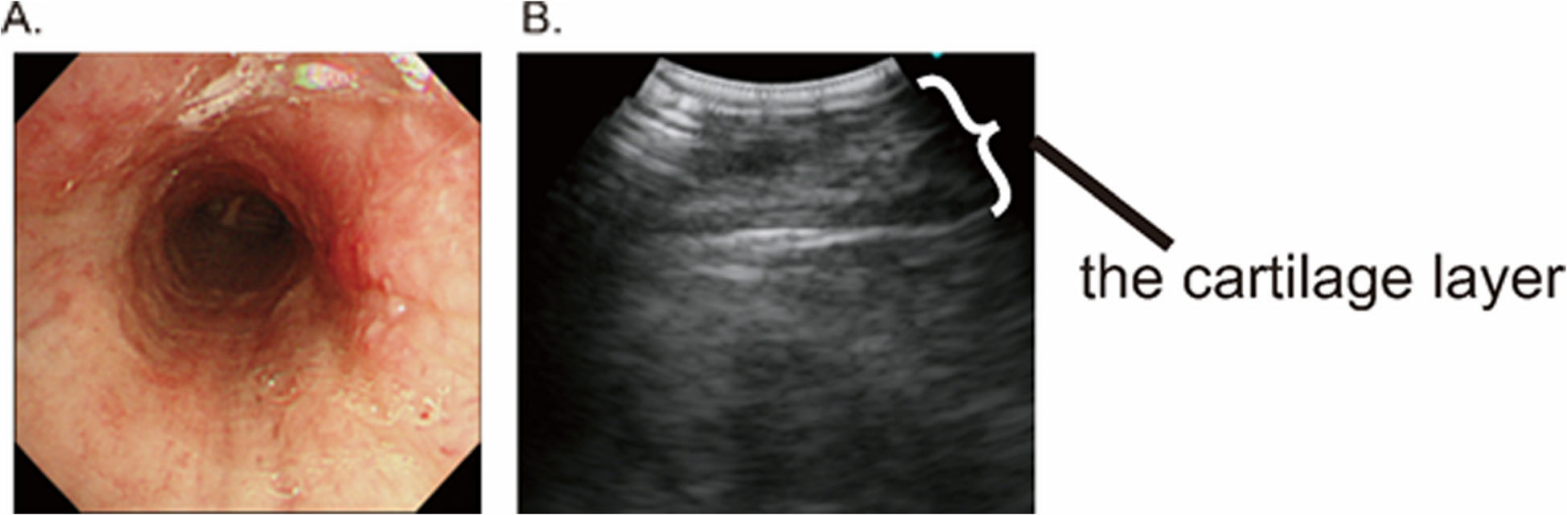
Imaging findings of bronchoscopy. **a** Bronchoscopy shows narrowing of the trachea and the straight pattern on the membranous portion with disappearance of the tracheal cartilage. **b** Endobronchial ultrasonography (EBUS) of trachea shows thickening of the cartilage layer

**Fig. 4:**
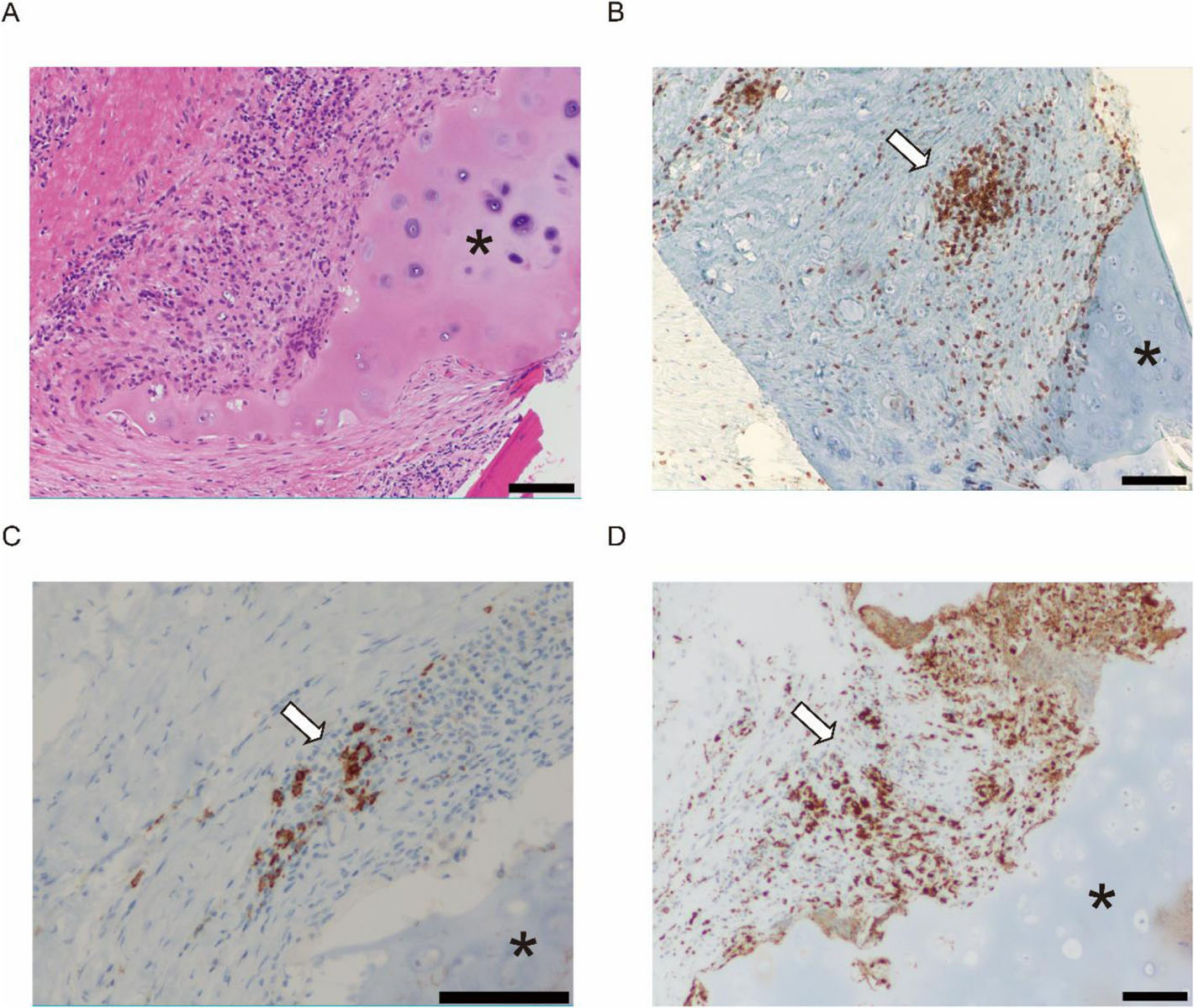
Histopathological findings of the surgical biopsy specimen from the trachea. **a** Histological examination by hematoxylin-eosin staining of the lesion shows infiltration of inflammatory cells with destruction of the cartilage. **b**-**d** Immunohistochemical staining of tracheal wall with anti-CD3 (**b**), anti-CD20 (**c**), or anti-CD68 (**d**) antibody. Arrow represents CD3-, CD20- or CD68- positive cells, respectively. Magnifying power is (×100) except for (C) (×200). Asterisks represent tracheal cartilage. Bar, 100 μm

A 62-year-old man who had systemic sclerosis presented with cough and dyspnea on exertion. Lung function test showed an expiratory flow limitation (Fig. 1) and chest computed tomography showed diffuse thickness from the trachea to the bronchiole (Fig. 2). No other tests showed abnormal findings and the level of C-reactive protein was normal. To evaluate further, bronchoscopy was performed and the endoscopic findings showed disappearance of the cartilaginous rings of the trachea and edematous changes of the tracheal membrane (Fig. 3a). Endobronchial ultrasonography (EBUS) showed thickening in the fourth-marginal echo layer, which suggested inflammation in the cartilage [9] (Fig. 3b). However, the tracheal biopsy showed no specific findings. Therefore, surgical biopsies from the trachea were obtained under general anesthesia. The histological examination showed inflammatory cell infiltration with destruction of the cartilage (Fig. 4a), and these inflammatory cells consisted of CD3-positive T-lymphocytes, CD20-positive B-lymphocytes and CD68-positive macrophages (Fig. 4b-d). In addition, there were no findings of vasculitis, granuloma or amyloid by Congo red staining (data not shown). The serum level of anti-type II collagen antibody, which is known to be elevated in RPC patients [10], was also elevated to a concentration of 98 EU/ml, while it is generally less than 25 EU/ml in normal subjects. At that time, the patient did not have auricular or nasal symptoms. However, we performed a biopsy from the left external ear because a previous report demonstrated the diagnostic usefulness of ear biopsy even if there are no auricular symptoms [11]. Nevertheless, the histological examination did not show any inflammatory changes in the auricular cartilage (data not shown). Finally, we diagnosed him as localized tracheobronchial RPC.

**Fig. 5:**
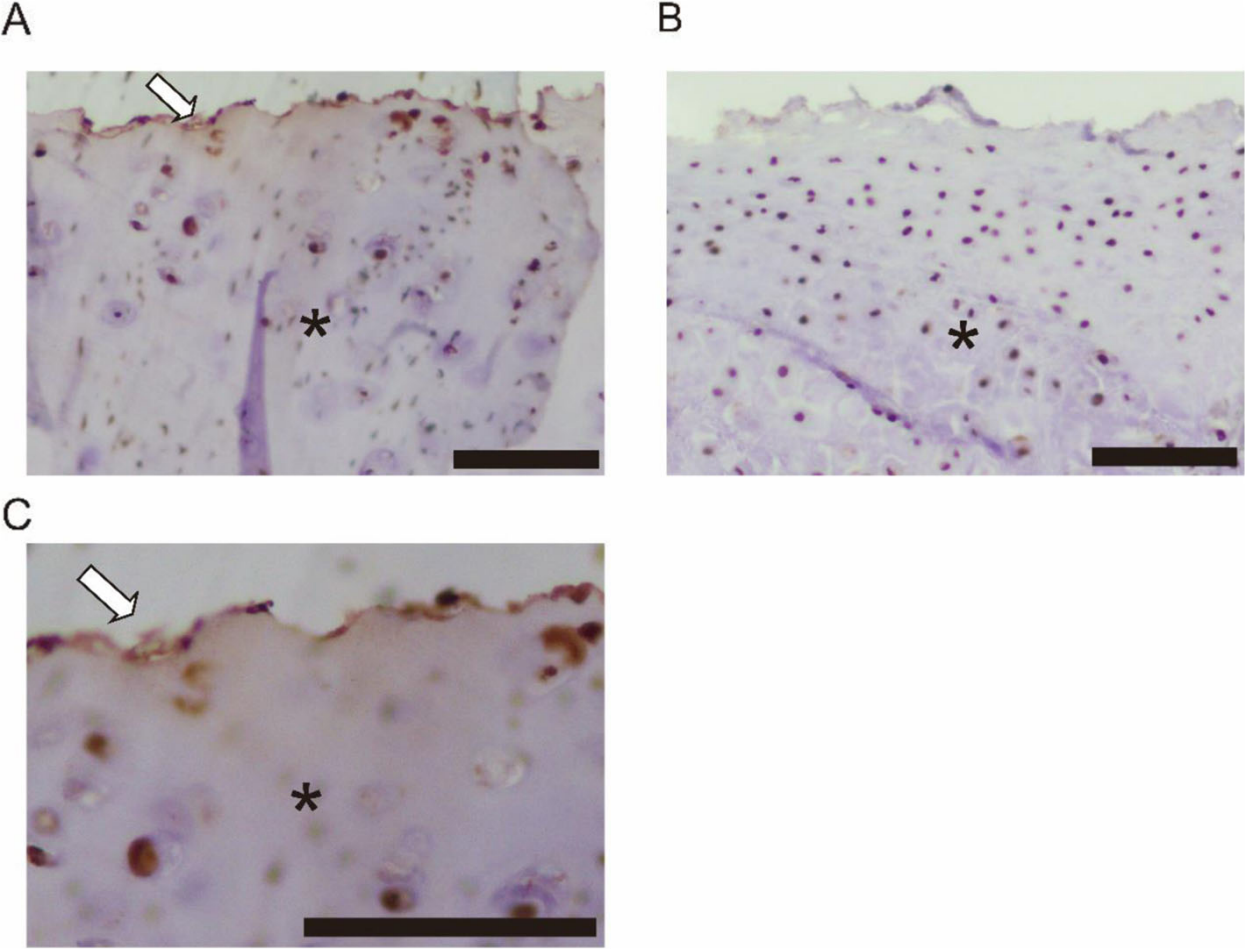
Immunohistochemical staining of matrilin-1 in the tracheal and auricular specimen. Immunohistochemical staining of tracheal and auricular cartilage with anti-matrilin-1 antibody. Immunoreactions are visualized with 3, 3-diaminobenzidine and counterstained with hematoxylin. Matrilin-1 immunoreactivity (brown, arrow) is detected mainly in the boundary region of the cartilage and some chondrocytes in the trachea (**a**, **c**), but not in the auricular tissue (**b**). Asterisk represents the cartilage. Magnifying power is (×100) both in (**a**) and (**b**). High-magnification image (×200) is shown in (**c**). Bar, 100 μm

To clarify the hypothesis that autoimmunity against matrilin-1 is involved in the pathogenesis of localized tracheobronchial RPC, we evaluated the expression level of matrilin-1 in tracheal and auricular specimens from this patient. We performed immunohistochemical staining of the tracheal and auricular tissues using antibodies against matrilin-1 (dilution: 1/100, Clone # 828806, R&D systems, Minneapolis, MN) after antigen retrieval by autoclaving the slides. As we expected, immunohistochemical staining with anti-matrilin-1 antibody showed that matrilin-1 immunoreactivity was detected mainly in the boundary region of cartilage in the tracheal (Fig. 5a, c), but not in the auricular cartilage (Fig. 5b).

## Discussion and conclusions

Relapsing polychondritis (RPC) is a rare systemic inflammatory disease affecting the cartilaginous structures, and diagnosis of RPC is based on criteria suggested by Michet, et al [1, 3, 12]. The criteria require confirmed inflammation in two of three auricular, nasal or laryngotracheal cartilage, or proven inflammation in one of the above types of cartilage and two other minor criteria including hearing loss, ocular inflammation, vestibular dysfunction, and seronegative arthritis. Among the affected organs, auricular chondritis is a hallmark of RPC because it develops in more than 90% of such patients [13]. Tracheobronchial involvement appears in nearly half of RPC patients and most of the RPC patients have more than one organ symptom besides that of the airway [2]. Localized tracheobronchial RPC is much rarer and the diagnosis is generally difficult because of the absence of typical auricular or nasal symptoms at the onset [14–16]. A prolonged undiagnosed period can sometimes result in life-threatening tracheomalacia, which cannot be cured once it is established [3]. Therefore, early diagnosis of this disease is crucial. Until now, endoscopic EBUS and positron-emission tomography have been reported to be useful to evaluate the airway involvement of RPC patients [9, 16, 17]. However, as a differential diagnosis for localized tracheobronchial RPC, there remain other diseases, such as granulomatosis with polyangiitis, sarcoidosis and amyloidosis [15, 18]. In the present case, there was no sign of cartilaginous inflammation except for airway symptoms. Although endoscopic and EBUS findings and increased levels of serum anti-type II collagen antibody suggested RPC in this patient, we finally confirmed the diagnosis with histological examination of surgical tracheal biopsies.

Occasionally, RPC has been known to coexist with other autoimmune diseases [2, 19]. Antinuclear antibodies or anti-neutrophil cytoplasmic antibodies have been also reported to be present in RPC patients. In the present case, the patient suffered from systemic sclerosis for a long time before the onset of RPC. This background suggested the presence of an autoimmunity contribution to the onset.

At present, some types of cartilage antigens are presumed to be involved in the RPC pathogenesis. One of the candidates is type II collagen, which consists of 95% of the total collagen in cartilage, and anti-type II collagen antibody is detected in one third of RPC patients, the same as in rheumatoid arthritis [10, 20]. In the present case, anti-type II collagen antibody was in fact elevated. However, type II collagen seems not to explain the pathogenesis of localized tracheobronchial RPC because this collagen is expressed in other types of cartilage such as articular cartilage. On the other hand, a non-collagenous cartilage matrix protein, matrilin-1, is known to be abundantly expressed in tracheal cartilage, but less so in articular cartilage in bovine and mice [4]. In human, the serum level of matrilin-1 has been shown to be higher during the active phase in RPC patients [21] and an increased level of anti-matrilin-1 antibodies has been also detected in 13.4% of RPC patients [8]. In the present case, we did not measure the serum level of matrilin-1 and anti-matrilin-1 antibodies because there was no serum sample left and no means of measuring the level commercially. In an animal model, immunization with matrilin-1 has been demonstrated to cause severe respiratory dysfunction in mice and rats [5, 6]. Therefore, autoimmunity against the matrilin-1 has been assumed to be involved in the pathogenesis of RPC with respiratory symptoms. However, until now, there has been no study that verified the expression of matrilin-1 in the cartilage of localized tracheobronchial RPC patients, as far as we know. In RPC animal models, the expression of matrilin-1 has been demonstrated on the surface of tracheal cartilage [6, 8]. As is the case with RPC animal models, this patient showed the expression of matrilin-1 in the boundary region of cartilage and some chondrocytes in the trachea, but not in the auricular cartilage. Consistent with these findings, cartilage destruction in this patient was only seen in the trachea, not in auricular cartilage. This finding suggests that expression level of matrilin-1 and autoimmunity to this protein could be involved in the pathogenesis of localized tracheobronchial RPC in human. However, immunochemical staining of matrilin-1 in the tracheal tissue would not be useful for the diagnosis of RPC at present, because there has been no reported comparison of the expression levels between RPC patients and normal subjects. Further studies are needed to clarify this point.

In conclusion, we first demonstrated the expression of matrilin-1 in tracheal but not auricular cartilage in a patient with localized tracheobronchial RPC. This result supports the possibility that matrilin-1 could be involved in the pathogenesis of localized tracheobronchial RPC. However, this is only one case report and further observations will be needed to confirm this result.

## Data Availability

All the data supporting our findings is contained within the manuscript.

## Abbreviations

CT: Computer tomography
EBUS: Endobronchial ultrasonography
FEV_1_: Forced expiratory volume in 1 second
PEF: Peak expiratory flow
RPC: Relapsing polychondritis

## References

[1] Michet CJ, Mc KC, Luthra HS, O’Fallon W (1986). Relapsing polychondritis: survival and predictive role of early disease manifestations. Ann Intern Med.

[2] Cantarini L, Vitale A, Brizi MG, Caso F, Frediani B, Punzi L, Galeazzi M, Rigante D (2014). Diagnosis and classification of relapsing polychondritis. J Autoimmun.

[3] Borgia F, Giuffrida R, Guarneri F, Cannavo SP (2018). Relapsing Polychondritis: An Updated Review. Biomedicines.

[4] Klatt AR, Becker AK, Neacsu CD, Paulsson M, Wagener R (2011). The matrilins: modulators of extracellular matrix assembly. Int J Biochem Cell Biol.

[5] Hansson AS, Heinegard D, Holmdahl R (1999). A new animal model for relapsing polychondritis, induced by cartilage matrix protein (matrilin-1). J Clin Invest.

[6] Hansson AS, Johannesson M, Svensson L, Nandakumar KS, Heinegard D, Holmdahl R (2004). Relapsing polychondritis, induced in mice with matrilin 1, is an antibody- and complement-dependent disease. Am J Pathol.

[7] Saxne T, Heinegard D (1995). Serum concentrations of two cartilage matrix proteins reflecting different aspects of cartilage turnover in relapsing polychondritis. Arthritis Rheum.

[8] Hansson A-S, Heinegård D, Piette J-C, Burkhardt H, Holmdahl R (2001). The occurrence of autoantibodies to matrilin 1 reflects a tissue-specific response to cartilage of the respiratory tract in patients with relapsing polychondritis. Arthritis Rheum.

[9] Miyazu Y, Miyazawa T, Kurimoto N, Iwamoto Y, Ishida A, Kanoh K, Kohno N (2003). Endobronchial ultrasonography in the diagnosis and treatment of relapsing Polychondritis with tracheobronchial Malacia. CHEST.

[10] Aigner T, Stove J (2003). Collagens-major component of the physiological cartilage matrix, major target of cartilage degeneration, major tool in cartilage repair. Adv Drug Deliv Rev.

[11] Jung C, Muller-Hocker J, Rauh G (1996). Relapsing poly (peri) chondritis diagnosed by biopsy during inflammatory free interval: destructive polychondritis versus fibrosing perichondritis. Eur J Med Res.

[12] McAdam LP, O'Hanlan MA, Bluestone R, Pearson CM (1976). Relapsing polychondritis: prospective study of 23 patients and a review of the literature. Medicine.

[13] Arnaud L, Mathian A, Haroche J, Gorochov G, Amoura Z (2014). Pathogenesis of relapsing polychondritis: a 2013 update. Autoimmun Rev.

[14] Tsunezuka Y, Sato H, Shimizu H (2000). Tracheobronchial involvement in relapsing Polychondritis. Respiration.

[15] Maimon N, Marras T, Hwang D, Paul N, Keshavjee S, Chan CK (2006). A 46-year-old female with dyspnoea, stridor and chronic cough. Eur Respir J.

[16] Suzuki S, Ikegami A, Hirota Y, Ikusaka M (2015). Fever and cough without pulmonary abnormalities on CT: relapsing polychondritis restricted to the airways. Lancet.

[17] De Geeter F, Vandecasteele SJ (2008). Fluorodeoxyglucose PET in relapsing polychondritis. N Engl J Med.

[18] Ingegnoli A, Corsi A, Verardo E, De Filippo M, Sverzellati N, Zompatori M (2007). Uncommon causes of tracheobronchial stenosis and wall thickening: MDCT imaging. La Radiologia medica.

[19] Frances C, el Rassi R, Laporte JL, Rybojad M, Papo T, Piette JC (2001). Dermatologic manifestations of relapsing polychondritis. A study of 200 cases at a single center. Medicine.

[20] Wooley PH, Luthra HS, O'Duffy JD, Bunch TW, Moore SB, Stuart JM (1984). Anti-type II collagen antibodies in rheumatoid arthritis. The influence of HLA phenotype. Tissue Antigens.

[21] Saxne T, Heinegård D (1989). Involvement of nonarticular cartilage, as demonstrated by release of a cartilage-specific protein, in rheumatoid arthritis. Arthritis Rheum.

